# Neuropathology and molecular diagnosis of Synucleinopathies

**DOI:** 10.1186/s13024-021-00501-z

**Published:** 2021-12-18

**Authors:** Shunsuke Koga, Hiroaki Sekiya, Naveen Kondru, Owen A. Ross, Dennis W. Dickson

**Affiliations:** Department of Neuroscience, Mayo Clinic, 4500 San Pablo Road, FL 32224 Jacksonville, USA

**Keywords:** Lewy body disease, Parkinson’s disease, Dementia with Lewy bodies, Multiple system atrophy, cryo-EM structures, RT-QuIC, PMCA, Biomarkers, AlphaFold

## Abstract

Synucleinopathies are clinically and pathologically heterogeneous disorders characterized by pathologic aggregates of α-synuclein in neurons and glia, in the form of Lewy bodies, Lewy neurites, neuronal cytoplasmic inclusions, and glial cytoplasmic inclusions. Synucleinopathies can be divided into two major disease entities: Lewy body disease and multiple system atrophy (MSA). Common clinical presentations of Lewy body disease are Parkinson’s disease (PD), PD with dementia, and dementia with Lewy bodies (DLB), while MSA has two major clinical subtypes, MSA with predominant cerebellar ataxia and MSA with predominant parkinsonism. There are currently no disease-modifying therapies for the synucleinopathies, but information obtained from molecular genetics and models that explore mechanisms of α-synuclein conversion to pathologic oligomers and insoluble fibrils offer hope for eventual therapies. It remains unclear how α-synuclein can be associated with distinct cellular pathologies (e.g., Lewy bodies and glial cytoplasmic inclusions) and what factors determine neuroanatomical and cell type vulnerability. Accumulating evidence from *in vitro* and *in vivo* experiments suggests that α-synuclein species derived from Lewy body disease and MSA are distinct “strains” having different seeding properties. Recent advancements in *in vitro* seeding assays, such as real-time quaking-induced conversion (RT-QuIC) and protein misfolding cyclic amplification (PMCA), not only demonstrate distinct seeding activity in the synucleinopathies, but also offer exciting opportunities for molecular diagnosis using readily accessible peripheral tissue samples. Cryogenic electron microscopy (cryo-EM) structural studies of α-synuclein derived from recombinant or brain-derived filaments provide new insight into mechanisms of seeding in synucleinopathies. In this review, we describe clinical, genetic and neuropathologic features of synucleinopathies, including a discussion of the evolution of classification and staging of Lewy body disease. We also provide a brief discussion on proposed mechanisms of Lewy body formation, as well as evidence supporting the existence of distinct α-synuclein strains in Lewy body disease and MSA.

## 1. Introduction

Synucleinopathies are a group of neurodegenerative disorders characterized by neuronal or glial inclusions, or both, composed of aggregated α-synuclein. Pathologically, synucleinopathies can be divided into two major disease groups: Lewy body disease and multiple system atrophy (MSA) [1–3]. Lewy body disease is a pathologic term for neurodegenerative disorders characterized by aggregated α-synuclein in perikarya and neurites of neurons (Lewy bodies and Lewy neurites) [4]. Lewy body pathology is not restricted to Parkinson’s disease (PD), PD dementia (PDD), and dementia with Lewy bodies (DLB), but is also observed in several neurodevelopmental and neurometabolic disorders, such as *PLA2G6*-associated neurodegeneration (i.e., infantile neuroaxonal dystrophy, atypical neuroaxonal dystrophy, adult-onset dystonia-parkinsonism, and autosomal recessive early-onset parkinsonism), *POLG*-associated neurodegeneration, Niemann-Pick type C1, and Krabbe disease [5]. Although these diseases have α-synuclein aggregates at a much younger age than incidental Lewy bodies observed in other neurodegenerative disorders or healthy elderly individuals, these disorders are not included in this review. We herein describe clinical, genetic, and neuropathologic features of synucleinopathies, including a historical evolution of the disease concept, classification, and staging of Lewy body disease. We also review evidence supporting the existence of distinct α-synuclein strains in Lewy body disease and MSA, with a specific focus on *in vitro* seeding assays and cryogenic electron microscopy (cryo-EM) structural studies of α-synuclein.

## 2. Background

### 2.1 History of synucleinopathies

James Parkinson published a short monograph, “An Essay on Shaking Palsy,“ in 1817 [6], in which he described six patients with tremors, slow movements, and falls. His work received little attention for decades until Jean-Martin Charcot introduced it in his lectures in the late 1800’s. Charcot added rigidity as a prominent symptom and pointed out that the disability is not caused by palsy; he proposed to call the disease PD. In 1912, Friedrich H. Lewy reported the presence of intracellular inclusion bodies in the dorsal nucleus of the vagus nerve, nucleus basalis of Meynert, and the thalamic paraventricular nucleus of the brains of PD patients. Gonzalo Rodriguez Lafora also examined one patient with PD and confirmed the existence of neuronal inclusions in 1913. Konstantin Tretiakoff reported neuronal loss and neuronal inclusions in the substantia nigra in 1919, and it was proposed that these inclusions be called Lewy bodies [7–9]. In 1961, Haruo Okazaki reported two patients with progressive dementia without parkinsonism who had abundant Lewy bodies in the neocortex [10]. Kenji Kosaka subsequently found similar inclusions in the neocortex of patients with cognitive impairment and extrapyramidal symptoms and proposed the term diffuse Lewy body disease for this disorder [11]. Subsequently, an international consortium led by Ian McKeith coined the term DLB to refer to this clinicopathologic disorder [12]. Lewy bodies are now considered a pathological hallmark of PD, DLB, and PDD.

MSA is a distinctive synucleinopathy that includes two major clinicopathologic subtypes previously thought to be separate disorders: olivopontocerebellar atrophy (OPCA) and striatonigral degeneration (SND). The term Shy-Drager syndrome was originally used in 1960 to describe a neurological disorder associated with orthostatic hypotension of unknown etiology, but the pathologic descriptions by Shy and Drager clearly indicated that it was a disorder affecting multiple systems beyond the autonomic nervous system [13]. Accumulating evidence indicates that the majority of patients with SND have some degree of OPCA and vice versa. Moreover, progressive autonomic failure corresponding to Shy-Drager syndrome is common in both SND and OPCA. Therefore, Graham and Oppenheimer considered these variants to be the same disorder and coined the term MSA in 1969 [14]. Although there had been some debate about whether MSA was a single disease, the discovery of argyrophilic glial cytoplasmic inclusions (GCI), also known as Papp-Lantos bodies [15, 16], in SND and OPCA provided strong evidence that they were part of a disease spectrum [16].

Seminal studies in the early 1990’s in the laboratory of Tsunao Saitoh discovered a non-amyloid component in senile plaques (NACP) [17] using biochemical methods, and later showed that NACP was a synaptic protein with genetic locus on chromosome 4q21.3-q22 identical to α-synuclein [18]. Previous studies had defined α-synuclein as one member of a family of presynaptic proteins (α-, β- and γ-synuclein) with a role in membrane-associated processes at the presynaptic terminal (reviewed in [19]). In 1997, Polymeropoulos discovered a mutation in the gene for α-synuclein (*SNCA*) in Southern Italian families of Greek heritage with PD [20]. Subsequently, Spillantini and colleagues reported that α-synuclein was the major component of Lewy bodies in sporadic PD and DLB patients [21]. These findings combined pathological and genetic insight and established the disease concept of “synucleinopathy.” Wakabayashi and colleagues reported that the major protein component in GCI was also α-synuclein, linking MSA and Lewy body disease as the two major clinicopathologic subtypes of synucleinopathy [22].

### 2.2 Clinical spectrum

Lewy body disease falls into three major clinicopathologic subtypes: PD, PDD, and DLB. PD is the most common neurodegenerative movement disorder characterized by bradykinesia, rigidity, rest tremor, and postural instability, as well as various non-motor symptoms, such as rapid eye movement sleep behavior disorder (RBD), hyposmia, depression, and autonomic failure [23]. Levodopa-responsiveness is a supporting feature of PD, and a feature that is useful in differentiating PD from atypical parkinsonian syndromes. Although there are differences between studies, up to 83% of patients with PD eventually develop dementia later in the disease course, which is termed as PDD [24–26]. DLB is the second most common cause of neurodegenerative dementia, after Alzheimer’s disease. The cardinal features of DLB include dementia, fluctuating cognition, visual hallucinations, RBD, and parkinsonism [27]. Motor symptoms may be absent in up to 25% of autopsy-confirmed DLB patients [28]. A clinical diagnosis of PDD or DLB requires information about not only the nature of clinical findings, but also the timing of dementia relative to motor signs and symptoms. Patients with PD who develop dementia later in the disease course (“more than one-year” after onset of parkinsonism) are considered to have PDD. On the other hand, patients who develop dementia, with (or without) parkinsonism after one year of cognitive or psychiatric symptoms are diagnosed with DLB [27]. In rare cases, patients with Lewy body disease present with focal cortical syndromes [29], such as corticobasal syndrome [30], progressive aphasia [31–33], or Capgras syndrome [34]. Although dysphagia is a common clinical feature of PD and atypical parkinsonian disorders [35–37], there are only a few reports of isolated dysphagia without extrapyramidal syndrome in patients with Lewy body disease, which is referred to as Lewy body dysphagia [38, 39].

MSA is a movement disorder characterized by a variable combination of autonomic failure, levodopa-unresponsive parkinsonism, cerebellar ataxia, pyramidal signs, and non-motor symptoms [37]. MSA is subclassified into two clinical subtypes depending upon the predominant motor symptom: MSA with predominant cerebellar ataxia (MSA-C) and MSA with predominant parkinsonism (MSA-P) [37]. Autonomic failure, such as urinary incontinence and orthostatic hypotension, is required for the clinical diagnosis of MSA. Besides autonomic failure, non-motor symptoms, such as RBD, sleep-disordered breathing, dysphagia, severe dysphonia, and dysarthria, are also common in MSA [37, 40, 41]. Unlike PDD and DLB, dementia is not a typical feature of MSA [37]; however, a subset of MSA patients develops cognitive deficits, particularly executive dysfunction [42–44].

RBD is a parasomnia characterized by dream-enacting behaviors due to lack of muscle atonia during REM sleep (“dream sleep”) [45]. This results in the common description of patients “acting out their dreams.” RBD is one of the most frequent nonmotor symptoms in both Lewy body disease and MSA. The absence of RBD after a 5-year disease duration is considered a “red flag” for the diagnosis of PD [23]. In the diagnostic criteria for DLB, RBD is one of the four core clinical features [27]. Although RBD is not included in current clinical criteria for MSA [37], several studies have supported its usefulness for the diagnosis of MSA [46–48]. In synucleinopathies, RBD often precedes motor or other nonmotor symptoms. Several longitudinal studies have demonstrated that up to 80% of patients with isolated RBD eventually develop clinical features of synucleinopathies, including PD, DLB, or MSA [49–51]. Therefore, isolated RBD has been considered a prodromal phase of synucleinopathies.

### 2.3 Pathogenesis of Lewy body formation

**Fig. 1:**
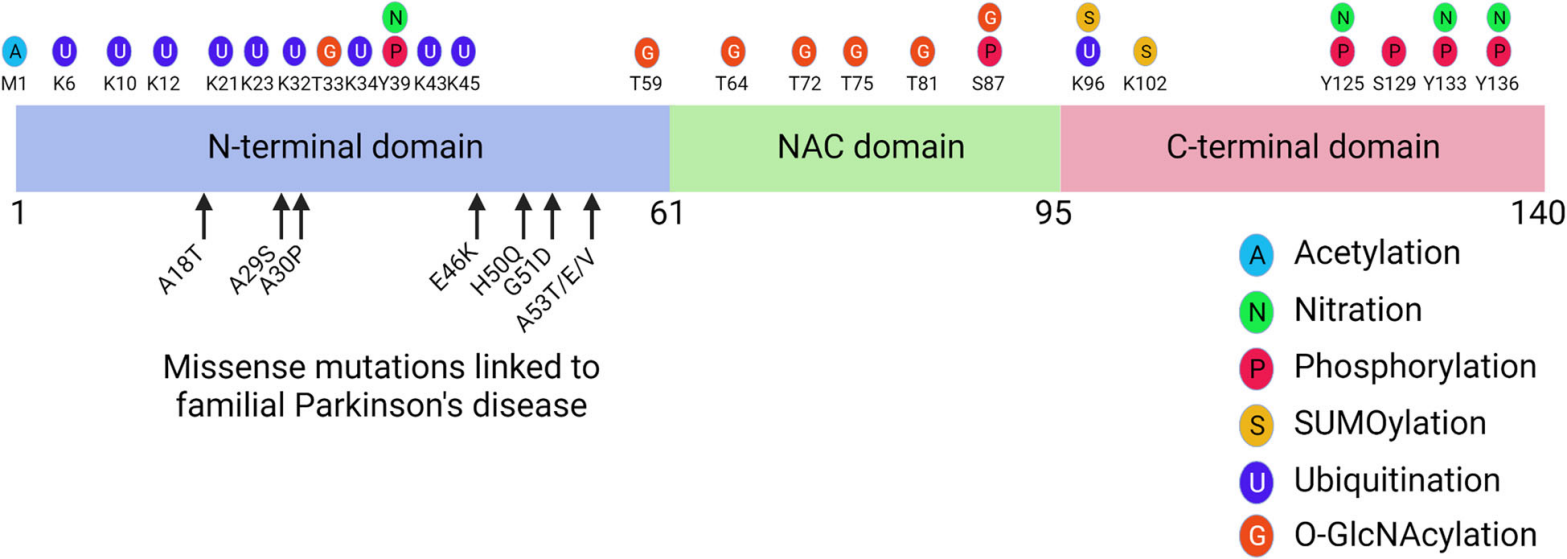
Structure of α-synuclein. Missense mutation sites linked to familial Parkinson’s disease and major post-translational modification sites are shown

α-Synuclein is a 140 amino acid protein that is encoded by the *SNCA* gene on chromosome 4q21 [52]. α-Synuclein consists of 3 modular domains (Fig. 1). The N-terminal amphipathic region (1-60 residues) of α-synuclein has lipid-binding properties [53]. The central region of α-synuclein contains a highly amyloidogenic domain, known as the non-amyloid-β component (NAC) domain (61-95 residues), which is critical for α-synuclein aggregation [17, 54]. The C-terminal acidic domain (96-140 residues) contributes to its natively unfolded structure. Truncation of this region results in increased potential for aggregation [55].

**Fig. 2:**
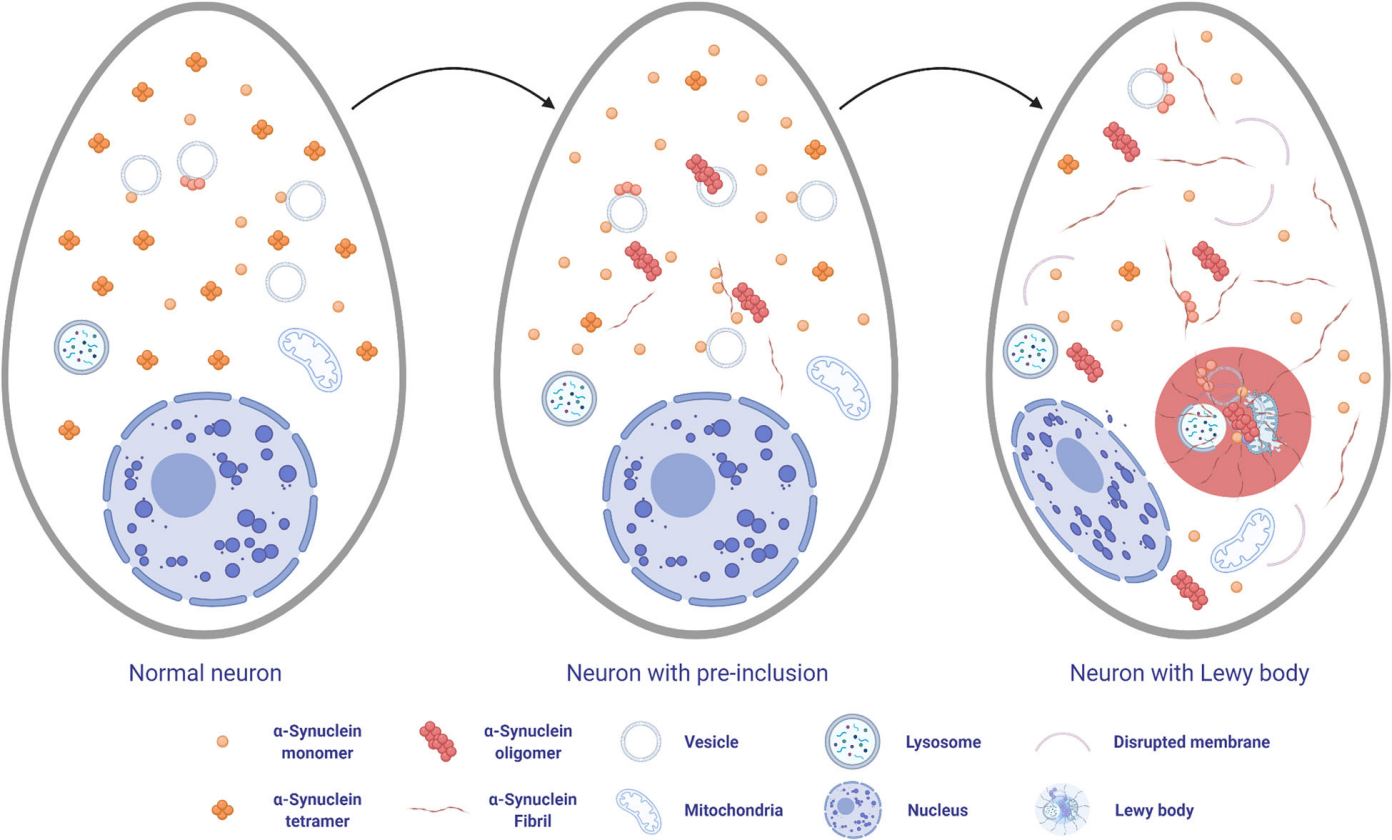
Hypothetical scheme of Lewy body formation in neurons. α-Synuclein exists in equilibrium between monomers and tetramers in the cytoplasm. Under pathologic conditions, the tetramer-monomer ratio decreases, and monomers bind to vesicle membranes. On the surface of membrane, α-synuclein tend to oligomerize, and toxic oligomers can disrupt lipid membranes. Monomers and oligomers form insoluble fibrils in the cytoplasm. Disrupted membranes, organelles, α-synuclein oligomers and fibrils are involved in the Lewy body formation

α-Synuclein is a neuronal protein, predominantly located in the presynaptic terminal [17]. Although the function of α-synuclein has not been fully elucidated, it is thought to play a role in regulating neurotransmitter release, synaptic function, and synaptic plasticity [56]. Under physiological conditions, α-synuclein in the cytoplasm of the neurons exists in a dynamic equilibrium between soluble monomers and a variety of oligomers, including helically folded tetramers (Fig. 2) [57, 58]. Although the process of Lewy body formation is not fully understood, binding of α-synuclein to lipid membranes is thought to be a key mediator of oligomerization and aggregation of α-synuclein [59–62]. Lipid membranes can promote α-synuclein aggregation, and α-synuclein oligomer species result in membrane permeabilization, which can disrupt membrane integrity [60]. Destabilization of stable α-synuclein tetramers is also considered an early step in aggregation of α-synuclein [63]. Indeed, mutations in *SNCA* associated with familial PD or exposure to unsaturated fatty acids, such as oleic acid, decrease the tetramer-monomer ratio and induce cytoplasmic inclusions in human cellular models [64, 65]. Loss of nuclear membrane integrity may also trigger α-synuclein aggregation through interaction between α-synuclein and nuclear proaggregant factors, such as histone [66, 67]. A recent study using stimulated emission depletion (STED)-based super-resolution microscopy revealed that Lewy bodies contain not only aggregates of α-synuclein, but also crowded organelles and lipid membranes, including damaged lysosomes, mitochondria, and various vesicles (Fig. 2) [68].

α-Synuclein undergoes various post-translational modifications, including phosphorylation, ubiquitination, acetylation, nitration, O-GlcNAcylation and SUMOylation (Fig. 1) [69, 70]. Post-translational modifications of α-synuclein may influence its toxicity and propensity to aggregate. Phosphorylation of α-synuclein is the most investigated post-translational modification of α-synuclein, and it is considered a marker of pathologic α-synuclein [71]. Up to 90% of α-synuclein is phosphorylated at serine-129 in Lewy bodies, while only 4% of soluble α-synuclein is phosphorylated at this residue [71]. Multicolor STED microscopy revealed the subcellular arrangement of α-synuclein in Lewy body [72], with serine-129-phosphorylated α-synuclein at the periphery of Lewy body with cytoskeletal proteins, while α-synuclein truncated at aspartate-119 or asparagine-122 was condensed in the Lewy body core [72].

## 3. Genetics

### 3.1 Genetics of Lewy body disease

A direct causal relationship between α-synuclein and PD was first established in 1997 with the discovery of a single point mutation in the *SNCA* gene resulting in a non-synonymous amino acid substitution, p.A53T, causing familial early-onset PD [20]. Subsequently, several other substitutions in the α-synuclein protein (e.g., p.A18T, p.A29S, p.A30P, p.E46K, p.G46K, p.H50Q, p.G51D, and p.A53E) have been linked to autosomal dominant familial PD [73–78]. Furthermore, genomic multiplications (i.e., duplication and triplication) of *SNCA* cause familial PD with extramotor features, including dementia [79, 80]. These genetic studies suggested that overexpression of wild-type α-synuclein could be pathogenic and that the severity of the phenotype may be dose-dependent. For example, the clinical phenotypes tended to be more severe in families with *SNCA* triplication than *SNCA* duplication (e.g., non-motor features were more frequent in triplication families). Patients with *SNCA* triplication had greater severity of oligodendroglial and extra-perikaryal α-synuclein pathologies than in sporadic PD [81]. This finding suggests that high α-synuclein expression is associated with pathologic α-synuclein deposition in both neurons and oligodendrocytes. Importantly, this observation also nominated reduction of α-synuclein levels as a possible therapeutic strategy for synucleinopathies.

In addition to *SNCA*, pathogenic mutations in other genes have been associated with familial PD, including *LRRK2*, *VPS35*, *PRKN*, *PINK1*, *DJ-1*, and *VPS13C* [82, 83]. As DNA sequencing technologies have revolutionized population genetics over the last decade, an increasing number of other genes have been nominated for PD (e.g., *CHCHD2*, *LRP10*, *TMEM230*, *DNAJC13*), but these genetic loci lack definitive confirmation [83]. Even though the discovery of familial PD-related genes has contributed to dissecting the etiology of the disease (e.g., dysfunction of vesicle trafficking, lysosomal, and mitochondrial pathways) [84], they still only account for a relatively small proportion of the genetic risk in PD. A genetic hypothesis for PD is that the disease develops through a complex interplay of low penetrant genetic risk variants and unknown environmental determinants. Notably, only *SNCA* and a subset of *LRRK2* mutations are indisputably associated with Lewy body pathology. Based on data from the Mayo Clinic brain bank, 59% (30/51) of cases with *LRRK2* mutations had Lewy body disease, while the remaining *LRRK2* cases had tau or TDP-43 pathology or nonspecific substantia nigra degeneration (unpublished data). The majority of the genes nominated for familial PD (see above) do not have α-synuclein pathology, or there are inconsistent reports of Lewy body pathology in the small number of patients who have come to autopsy [83]. This fact indicates that “familial PD” in the context of most genetic studies may include a variety of disorders, including some that are not associated with Lewy body disease.

Population-based approaches to resolve the genetic architecture of PD have focused on common variant associations at candidate genes, including *SNCA* and *MAPT* loci. Large unbiased studies characterizing patterns of linkage disequilibrium (e.g., the HapMap project) across the genome and high throughput genome-wide genotyping platforms, which simultaneously genotype hundreds of thousands of common single nucleotide variants have been performed. Early genome-wide association studies (GWAS) confirmed associations at the *SNCA* and *MAPT* loci, and highlighted novel associations at *LRRK2* and *PARK16* loci as risk factors for PD in both European and Japanese clinical cohorts [85, 86]. The only GWAS in autopsy-confirmed Lewy body-associated PD nominated a locus in *PARK11* [87], a locus on chromosome 2q associated with familial PD [88].

Of note, the *MAPT* locus was identified as a risk locus for PD only in European cohorts, while the *BST1* locus was identified as a risk locus only in Japanese cohorts [85, 86]. Additional loci have been nominated in Eastern Asian populations with GWAS methods [89]. The latest PD GWAS in Caucasian populations nominated 90 independent loci covering 78 genomic regions [90], with the *SNCA* locus showing the strongest signal. Although less well-characterized or studied, recent GWAS and whole-genome sequencing efforts in Lewy body dementia (i.e., PDD and DLB) in Caucasian populations have nominated at least five loci, including three that are also risk loci for PD (*SNCA*, *GBA*, and *TMEM175*) and two other (*APOE* and *BIN1*) known risk loci for Alzheimer’s disease [91, 92]. These findings indicate that Lewy body dementia shares genetic risk factors of both Alzheimer’s disease and PD, as may be expected from its mixed amyloid and α-synuclein pathology.

Another important genetic determinant of susceptibility to both PD and Lewy body dementia is variation in the *GBA* gene [93, 94]. Recessive mutations in *GBA* result in a lysosomal storage disorder known as Gaucher’s disease; however, astute clinical observations by Sidransky and co-workers noted that heterozygous carriers were at increased risk of PD and Lewy body dementia [95]. *GBA* is a GWAS locus for both disorders, but also is an example of a gene that harbors variants of differing penetrance, with rare variants also driving disease risk. *GBA* is the only significant finding in a gene burden analysis in a whole-genome sequence study of Lewy body dementia [39]. The role that *GBA* susceptibility variants may play in MSA remains controversial [96, 97].

### 3.2 Genetics of MSA

MSA is considered a sporadic disease; only a few familial MSA cases have been reported [98]. Several small multiplex families from Japan nominated mutations of the *COQ2* gene for MSA [99]. In addition, a common substitution in *COQ2*, p.V393A, and several rare variants were associated with sporadic MSA [99]. The *COQ2* gene encodes an enzyme involved in synthesis of coenzyme Q10, and the p.V393A variant results in lower production of coenzyme Q10. Several studies from non-Asian countries, however, failed to replicate the findings [100, 101]. A meta-analysis of Eastern Asian populations confirmed an association of COQ2 p.V393A variant with MSA (odds ratio [OR] 2.05; 95% CI 1.29–3.25, p = 0.002) [102]. Interestingly, a subgroup analysis revealed that the association was significant for MSA-C (OR 2.75, 95% CI 1.98–3.84, p <0.001), but not for MSA-P (OR 1.25, 95% CI 0.64–2.46, p = 0.51). This finding may partially explain population variance since the predominant subtypes of MSA are different in Asian and Western countries. MSA-C is the predominant phenotype in Asia, while MSA-P is the predominant subtype in Western countries [103, 104]. Some studies reported decreased levels of coenzyme Q10 in the cerebellum of MSA, suggesting a potential association between alterations of coenzyme Q10 activity and cerebellar pathology [105, 106].

The largest GWAS, which enrolled 918 MSA patients of European ancestry and 3,864 controls, did not find any common genetic association [107]. This GWAS identified four potential risk loci, including single nucleotide polymorphisms in the genes *FBXO47*, *ELOVL7*, *EDN1*, and *MAPT*; however, these findings could not be replicated in a GWAS that included 906 MSA patients and 941 unrelated healthy controls of the Han Chinese population [108]. These discrepancies could be partially explained by ethnic differences, as GWAS demonstrated some differences in the genetic contribution to PD between the European and Asian populations [89]. Large-scale whole-genome sequencing efforts are underway in MSA, PD and Lewy body dementia, and it is likely that efforts of global consortia will be needed to fully resolve the role of genetics in synucleinopathies.

## 4. Pathology of synucleinopathies

### 4.1 Neuropathologic features of Lewy body disease

**Fig. 3:**
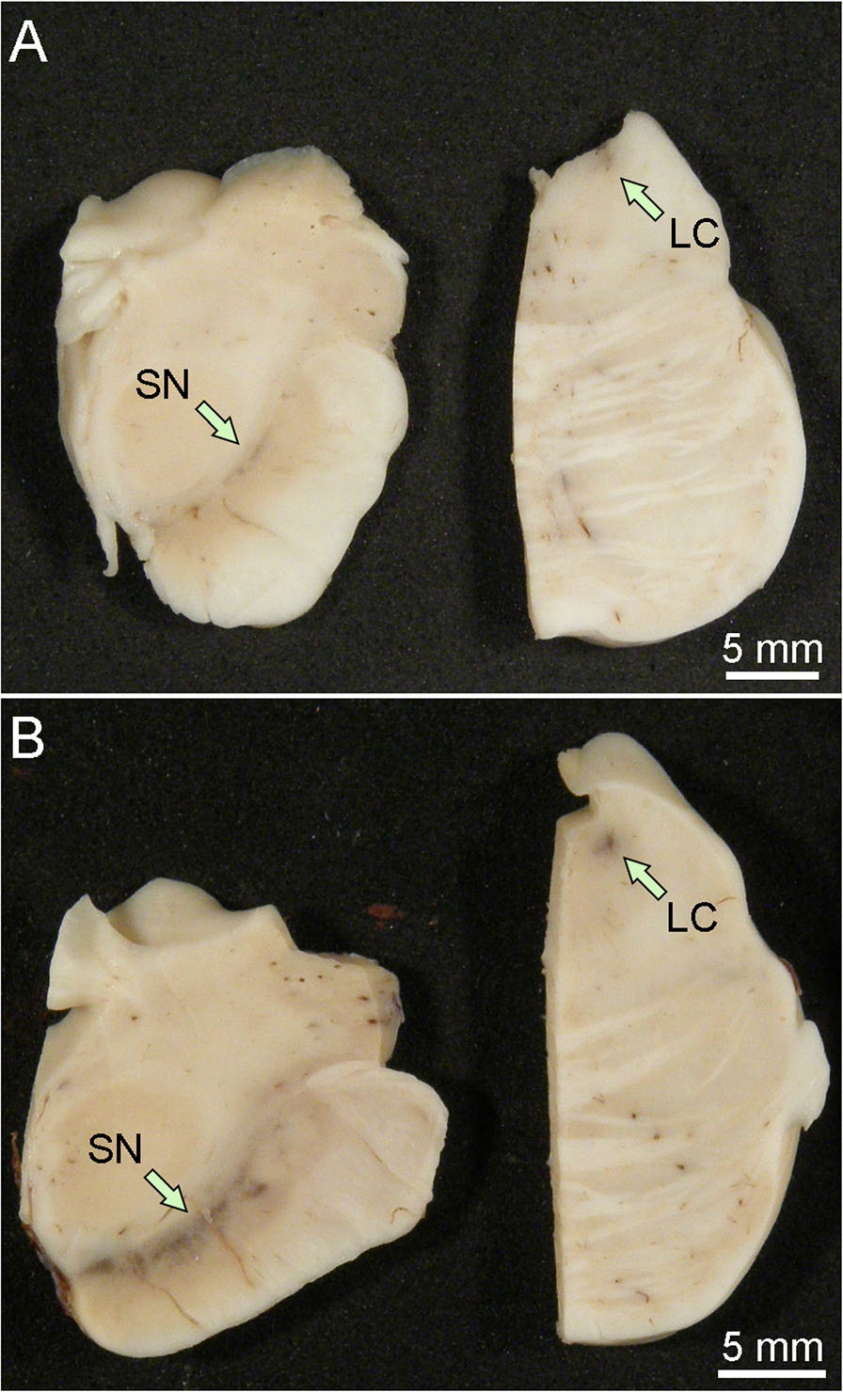
Macroscopic findings of Lewy body disease brains. The midbrain and pons from Lewy body disease (**A**) and control (**B**) brains. Loss of neuromelanin pigment in the substantia nigra and locus coeruleus is observed in Lewy body disease. Abbreviations: LC, locus coeruleus; SN, substantia nigra

In most cases of Lewy body disease without cognitive deficits, the macroscopic findings are comparable to age- and sex-matched controls, except for loss of neuromelanin pigment in the substantia nigra and locus coeruleus (Fig. 3). Dopaminergic neuronal loss in the substantia nigra, particularly in the ventrolateral part, is a pathologic hallmark of PD [109, 110]. The severity of neurodegeneration of the substantia nigra correlates with severity of extrapyramidal motor symptoms and the degree of striatal dopaminergic deficiency [111, 112]. Neuronal loss is moderate to marked in PD and PDD, but more variable in DLB. In fact, a subset of DLB patients lack parkinsonism and have preserved neuronal population in the substantia nigra. The locus coeruleus is a major noradrenergic nucleus, and neuronal loss leads to deficiency of noradrenaline, which may contribute to various symptoms, including cognitive impairment, affective symptoms, RBD, and gait difficulties [113].

**Fig. 4:**
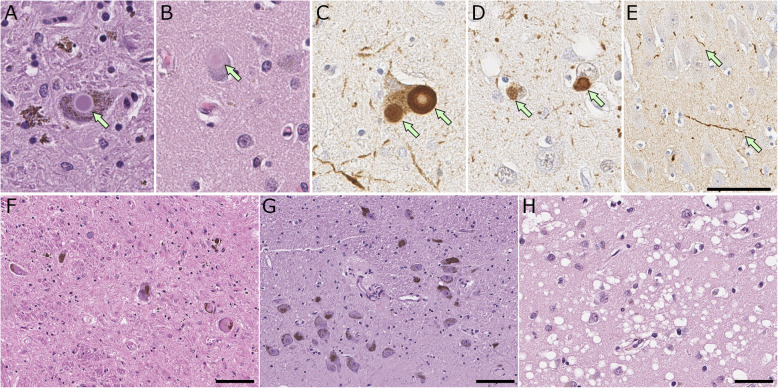
Representative images of histopathology of Lewy body disease. **A**, **B**, **F**-**H** hematoxylin and eosin staining, **C**-**E** immunohistochemistry for α-synuclein (NACP antibody). **A**, **C** Brainstem type Lewy body in the substantia nigra. **B**, **D** cortical type Lewy body (arrow) in the superior temporal cortex. **E** Lewy neurites in the CA2 sector of the hippocampus. **F**-**G** Lewy body disease shows neuronal loss with extracellular neuromelanin pigment in the substantia nigra (**F**), while it is minimum in a control case (**G**). **H** Spongiform change in the entorhinal cortex. Scale 100 μm in (**A**, **B**, and **H**); 50 μm in (**C**); 20 μm in (**D**-**G**, and **I**)

Lewy bodies are round, eosinophilic inclusions in neuronal perikarya. There are two types of Lewy bodies: classical (or brainstem) type and cortical type. Classical-type Lewy bodies have a dense hyaline appearance with a peripheral clear halo and are easily visible on hematoxylin and eosin stained sections (Fig. 4 A). Cortical type Lewy bodies have a less compact appearance and are more difficult to detect with histologic methods (Fig. 4B) [10]. Both types of Lewy bodies are strongly immunoreactive with antibodies to α-synuclein (Fig. 4 C, D) [71]. In addition to α-synuclein, more than 90 components of Lewy bodies have been reported, based mostly upon immunohistochemical colocalization with brainstem type Lewy bodies, including sequestration of neurotransmitter enzymes of cholinergic and dopaminergic neurons [114–116]. Accumulation of phosphorylated α-synuclein also occurs within cell processes (mostly axonal), so-called Lewy neurites (Fig. 4E). Lewy neurites in the CA2/3 sectors of the hippocampus are a characteristic histopathologic finding in many cases of PD and most cases of PDD and DLB [117]. Deposits of phosphorylated α-synuclein are observed less frequently in oligodendroglia, and rarely in astrocytes in the midbrain and basal ganglia [118].

In addition to Lewy bodies and neuronal loss in the substantia nigra (Fig. 4 F, G), a subset of cases have spongiform change or neuropil microvacuolation that is often most severe in the amygdala, but also seen in limbic and superior temporal cortices (Fig. 4 H) [119–121]. The most important co-pathology in Lewy body disease is Alzheimer-type pathology. In initial reports of diffuse Lewy body disease, the main neuropathologic features included not only numerous cortical Lewy bodies, but also numerous senile plaques and neurofibrillary tangles in the cerebral cortex [11]. The majority of Lewy body disease cases, not exclusively diffuse Lewy body disease, have some degree of Alzheimer-type pathology (i.e., neocortical senile plaques and neurofibrillary tangles) [122–125]. Senile plaques in the cerebral cortex are common, and they often are characterized by non-neuritic diffuse amyloid deposits [126]. Up to 50% of PDD cases and 28-89% of DLB cases have sufficient pathology for a secondary neuropathologic diagnosis of Alzheimer’s disease [127–129].

Lewy bodies are also found frequently in cases with advanced Alzheimer’s disease, particularly in the amygdala [130–134]. Hamilton screened α-synuclein pathology in 145 cases of Alzheimer’s disease and found that 88 cases (61%) had Lewy bodies [131]. Of regions screened, the amygdala was the most frequently affected region. Interestingly, some of the cases had numerous Lewy bodies in the amygdala, but minimal or no brainstem Lewy bodies (amygdala-predominant Lewy bodies). Uchikado and colleagues also screened Lewy-related pathology in 347 cases of Alzheimer’s disease and found that 62 cases (18%) were consistent with amygdala-predominant Lewy bodies [133]. Of those, Lewy bodies were only found in the amygdala (“amygdala-only” Lewy body disease) in 32 cases (9%). The clinical significance of amygdala-predominant Lewy bodies in Alzheimer’s disease remains uncertain [135], but evidence suggests that they may be associated with increased frequency of visual hallucinations compared to Alzheimer’s disease without amygdala Lewy bodies [136].

Unlike Lewy-related pathology, α-synuclein oligomers have not been possible to confidently detected with routine histologic methods. Roberts and colleagues applied proximity ligation assay to detect α-synuclein oligomers in histologic sections and showed α-synuclein oligomers in the cingulate cortex and reticular formation of the medulla in PD [137]. They also detected α-synuclein oligomers in morphologically intact neurons and in the periphery of Lewy bodies.

### 4.2 Classification and staging of Lewy body disease

The term “Lewy body disease” was coined by Kosaka and colleagues to refer to neurodegenerative diseases with numerous Lewy bodies in the central nervous system [4]. He classified Lewy body disease into three groups: diffuse, transitional and brainstem-predominant types [138]. Brainstem-predominant Lewy body disease corresponded to PD in their scheme, and diffuse Lewy body disease was thought to be an extension of PD pathology into the limbic lobe and the neocortex. Diffuse Lewy body disease was separated into two forms: a common form and pure form. The common form had not only numerous Lewy bodies, including neocortical Lewy bodies, but also many senile plaques and variable neurofibrillary tangles. In contrast, the pure form had few or no Alzheimer-type changes [122]. Kosaka later added a “cerebral type” of Lewy body disease, which had numerous Lewy bodies in the cerebral cortex and amygdala, but minimal or no Lewy bodies in the brainstem and diencephalon [139].

The First International Consortium for Lewy Body Dementia (ICDLB) proposed criteria for clinical and pathologic diagnosis of DLB [12]. Subtypes of Lewy-related pathology were categorized based upon the severity and topographical distribution of Lewy bodies [140]: diffuse neocortical, limbic (transitional), and brainstem-predominant, which corresponded roughly to Kosaka’s classification of diffuse, transitional and brainstem-predominant Lewy body disease. The Third ICDLB report developed a diagnostic scheme that was devised to predict the likelihood that the pathology would be associated with DLB. The criteria took into account both the extent of Lewy-related pathology and Alzheimer’s-type pathology to assign a probability that the pathology would be associated with the clinical presentation (Table 1). The severity of Lewy-related pathology was semi-quantitatively assessed on a five-point scale: 0 = none, 1 = mild, 2 = moderate, 3 = severe, and 4 = very severe. Recommended brain regions for assessment included the dorsal motor nucleus of vagus, locus coeruleus and substantia nigra in the brainstem, as well as nucleus basalis of Meynert, amygdala, transentorhinal cortex, cingulate cortex, temporal, frontal and parietal cortices. The likelihood of DLB clinical syndrome was directly related to severity of Lewy body pathology and indirectly related to severity of Alzheimer pathology. It was recognized from studies of prospective cohorts that when Alzheimer pathology was severe, most patients had Alzheimer type dementia, rather than DLB [141]. A recent study validates this approach in that the diagnostic sensitivity for probable DLB was significantly higher in limbic Lewy body disease and diffuse neocortical Lewy body disease without neocortical tangles than in those with neocortical tangles [142]. These findings indicate that the phenotypic expression of DLB is associated directly related to the extent of Lewy bodies and inversely related to the extent of neurofibrillary tangles [142].

**Table 1:** Likelihood of a typical clinical presentation of dementia with Lewy bodies

**Lewy-related pathology**	**ADNC based on NIA-AA**		
	**None/low**	**Intermediate**	**High**
Diffuse neocortical	High	High	Intermediate
Limbic (transitional)	High	Intermediate	Low
Brainstem-predominant	Low	Low	Low
Amygdala-predominant	Low	Low	Low
Olfactory bulb only	Low	Low	Low

*Abbreviations*: *ADNC *Alzheimer’s disease neuropathological change, *NIA-AA *National Institute on Aging–Alzheimer’s Association guidelines for the neuropathologic assessment of Alzheimer disease

Braak and colleagues proposed a staging scheme for Lewy-related pathology in PD [143] that was anatomically more detailed and specified than Kosaka’s classification of Lewy body disease. Lewy-related pathology initially occurred in the medulla oblongata (dorsal motor nucleus of vagus and the glossopharyngeal nucleus), and in the anterior olfactory nucleus of stage 1. In stage 2, α-synuclein pathology ascends to the pontine tegmentum, while stage 3 is associated with involvement of midbrain, stage 4 with limbic regions, and stages 5 and 6 with neocortical involvement. This staging scheme has largely been confirmed in several studies, but some exceptions have also been pointed out, such as cases with Lewy bodies restricted to the olfactory bulb or to the amygdala, particularly in cases with advanced Alzheimer’s disease (i.e., amygdala-predominant Lewy bodies). [133, 144, 145]. Following these studies, the ICDLB criteria were revised in 2017 to include amygdala-predominant and olfactory bulb types [27].

emi-quantitative evaluation has been used for diagnosing and classifying neurodegenerative disorders, but there are inherent weaknesses in semi-quantitative measures when it comes to inter-rater reliability [146]. Recently, Attems and colleagues proposed neuropathological consensus criteria for Lewy pathology, which they named “Lewy pathology consensus criteria” [147]. This new system is based on the ICDLB criteria modified to use a dichotomous approach for assessing Lewy-related pathology (i.e., present vs. absent), rather than semi-quantitative scores. In this scheme, a case that had very sparse Lewy bodies or Lewy neurites (i.e., only one Lewy neurite in a neocortical section) without Lewy-related pathology in other brain regions, such as medial temporal lobe, would be still assigned neocortical subtype. Nevertheless, in the select and relatively small study cohort, all cases with neocortical Lewy pathology had dementia. They also demonstrated that all cases were successfully classified, and that there was high inter-rater reliability. In parallel, they assessed other criteria on the same sections, and up to 30% of cases were unclassifiable with Braak staging or ICDLB criteria. Although this simple approach for Lewy pathology classification might be useful in routine diagnostic practice, clinicopathologic correlations of this classification, particularly neocortical type, need to be validated. They need to be compared critically to ICDLB neuropathologic criteria, which have been shown to “predict” the DLB clinical syndrome [148, 149].

### 4.3 Underlying pathology of PD, PDD, and DLB

PD, PDD, and DLB are considered distinct clinical entities that have varying degrees and distributions of Lewy body pathology. PD and PDD are disorders associated with extrapyramidal clinical syndrome of parkinsonism, which correlate less well with distribution of Lewy bodies, and better with nigrostriatal dopaminergic deficiency. Given this fact, ICDLB criteria were revised to capture this important clinical correlate, namely, moderate-to-severe neuronal loss in the ventrolateral substantia nigra [109]. The specification of the ventrolateral region was driven by the fact that ventrolateral dopaminergic neurons are the origin of the nigrostriatal dopaminergic pathway (projecting to the putamen and caudate nucleus), while ventromedial substantia nigra dopaminergic neurons are the origin of the mesolimbic pathway (projecting to the ventral striatum and nucleus accumbens).

While most patients with PD have brainstem-predominant Lewy body disease (or limbic Lewy body disease) at autopsy, a “pure form” of diffuse Lewy body disease can also be found in a few patients with PD [122, 150]. In contrast, most patients with PDD and DLB have diffuse Lewy body disease. It has proven challenging to differentiate DLB from PDD based on the neuropathologic findings alone [128]. Although DLB patients tend to have more severe Alzheimer-type pathology, and PDD patients tend to have a more severe neuronal loss in the substantia nigra [151], there are no clear neuropathologic distinctions between DLB and PDD, especially for patients with dementia and significant extrapyramidal signs [152].

### 4.4 Neuropathologic features and diagnosis of MSA

The current diagnostic criteria for MSA define three levels of certainty: possible, probable, and definite. The two former diagnoses are based on clinical features, while a definite diagnosis is based on neuropathologic evaluation [37]. A definite neuropathological diagnosis of MSA is established when there is evidence of widespread and abundant α-synuclein-positive GCI in association with neurodegenerative changes in striatonigral or olivopontocerebellar structures [153]. GCI are argyrophilic inclusions (positive on Gallyas silver stain) in the cytoplasm of oligodendrocytes [16]. They are visible on routine hematoxylin and eosin stains, but immunohistochemistry for phosphorylated α-synuclein is far more sensitive for visualization of GCI [22]. The pathogenesis of GCI is unknown, particularly the cellular origin of α-synuclein. Although oligodendrocytes express α-synuclein mRNA less than neurons [154, 155], mounting evidence from *in vitro* studies suggests that oligodendrocytes can take up α-synuclein monomers, oligomers, and fibrils, which are putatively involved in GCI formation [156–158].

**Fig. 5:**
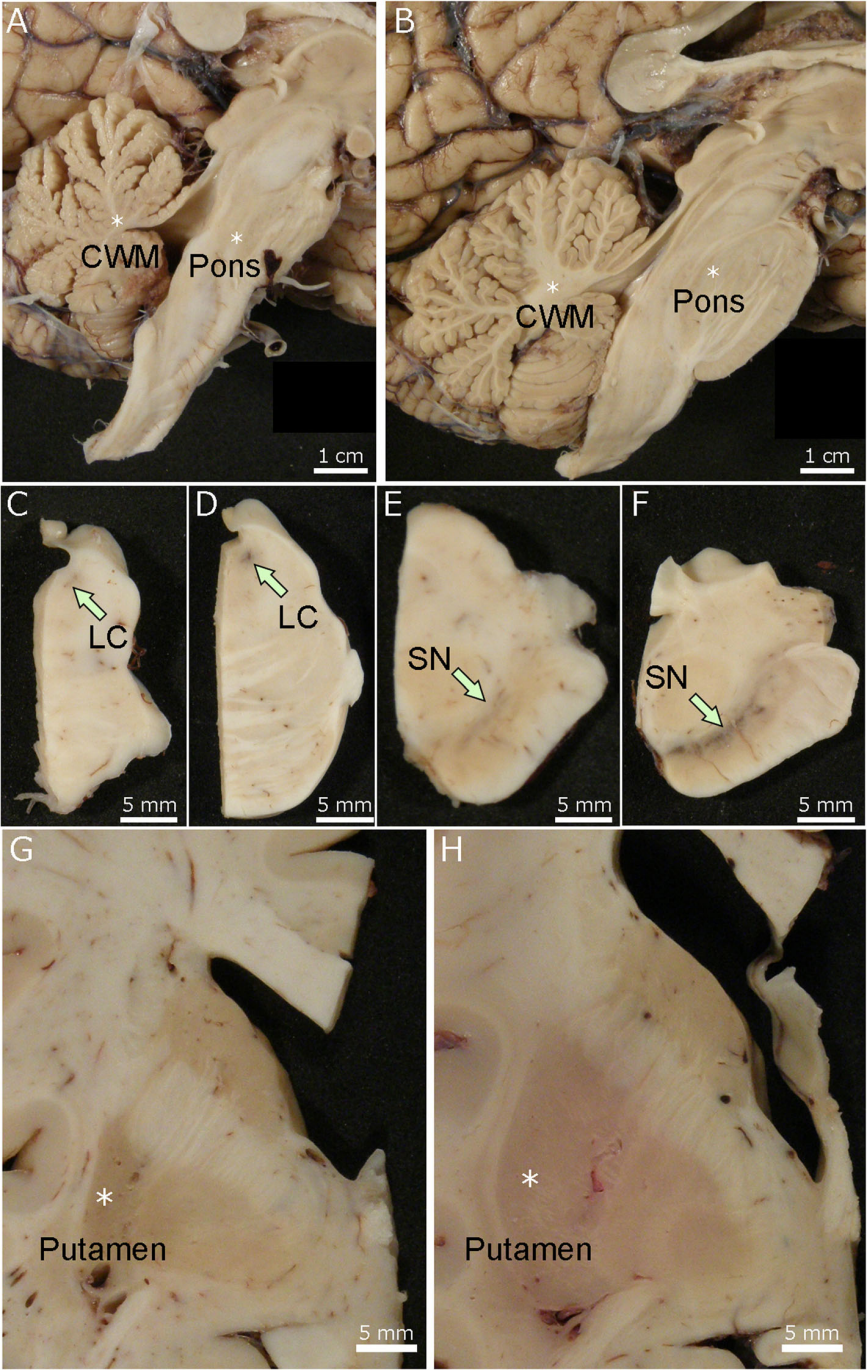
Macroscopic findings of multiple system atrophy (MSA). The brains from MSA (**A**, **C**, **E**, and **G**) and control (**B**, **D**, **F**, and **H**) cases. The pons and cerebellar white matter are atrophic in MSA (**A**). Loss of neuromelanin pigment in the locus coeruleus (**C**) and substantia nigra (**E**) is observed in MSA. The discoloration and atrophy of the lateral putamen in MSA (**G**). Abbreviations: CWM, cerebellar white matter; LC, locus coeruleus; SN, substantia nigra

Macroscopic findings in MSA vary with the subtype of MSA. In MSA-P, there is loss of neuromelanin pigment in the ventrolateral substantia, as well as atrophy and discoloration of the posterolateral putamen. In contrast, MSA-C has atrophy of the pontine base and the middle cerebellar peduncle with attenuation and discoloration of the cerebellar white matter (Fig. 5). The neocortex and limbic structures are usually macroscopically unremarkable.

**Fig. 6:**
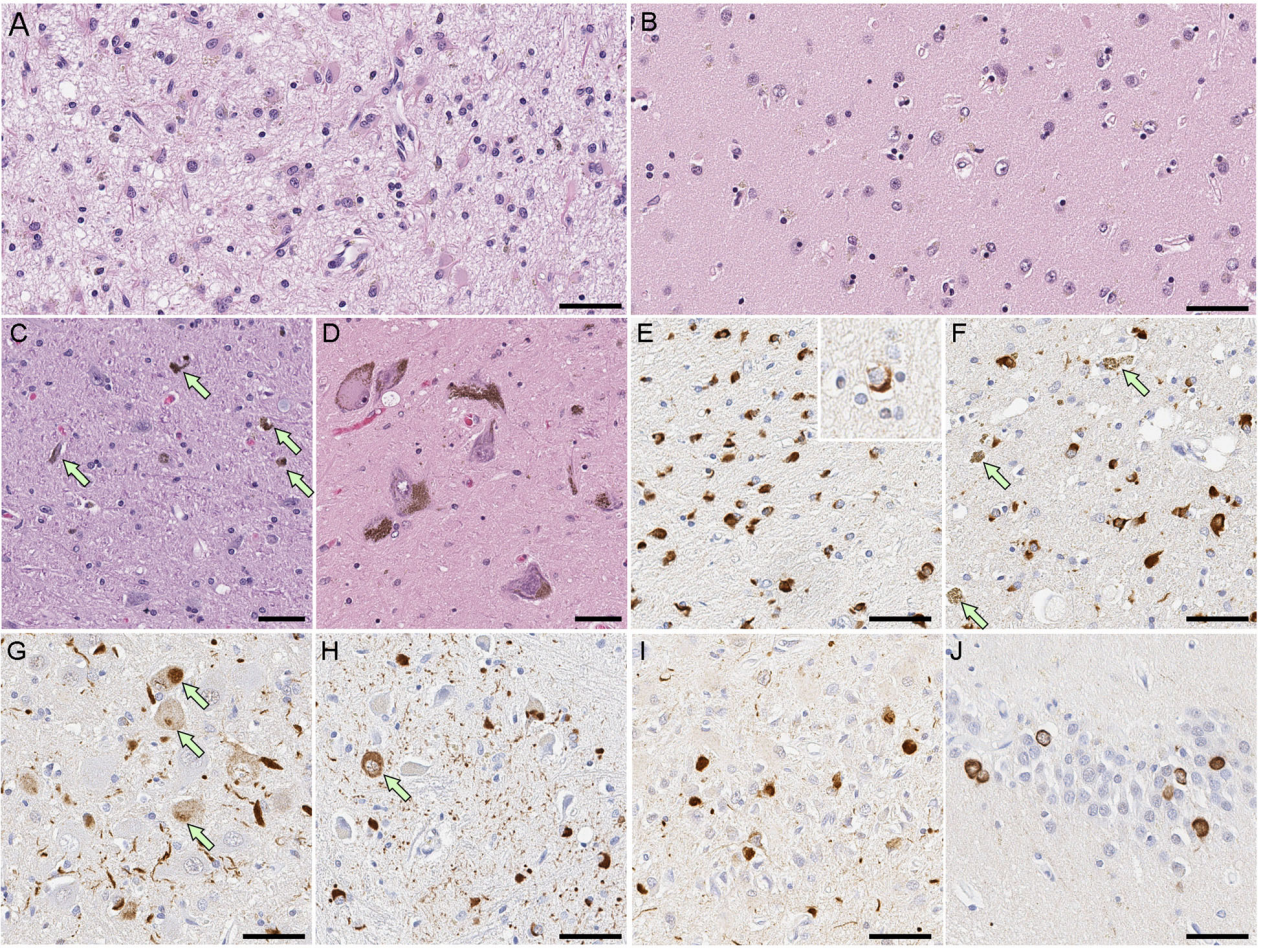
Representative images of histopathology of MSA. **A**-**D** Hematoxylin and eosin staining. **A** Severe neuronal loss with gliosis in the lateral putamen. **B** Neurodegeneration is minimal in the same region in a case of minimal change MSA. **C** A typical MSA shows neuronal loss with extracellular neuromelanin pigment (arrows) in the substantia nigra, while it is well preserved in minimal change MSA (**D**). **E**-**J** Immunohistochemistry for α-synuclein (NACP antibody). Numerous GCIs in the putamen (**E**) and substantia nigra (**F**). Arrows indicate extracellular neuromelanin pigment (**F**). Neuronal cytoplasmic inclusions in the pontine nucleus (**G**, arrows) and inferior olivary nucleus (**H**, arrow). Abundant neuronal cytoplasmic inclusions in the dentate fascia in FTLD-synuclein (**I**) and hippocampal MSA (**J**). Scale bars: 50 μm in all images

System-specific neuronal loss and gliosis are observed in both striatonigral and olivopontocerebellar systems (Fig. 6 A-D). In addition to these macroscopically affected brain regions, there are more widespread α-synuclein pathology, characterized by GCI (Fig. 6E, F) and variable neuronal cytoplasmic inclusions. The distribution of neuronal cytoplasmic inclusions is distinct from that of Lewy bodies, and they are most often observed in the putamen, pontine nuclei (Fig. 6G) and inferior olivary nuclei (Fig. 6 H), which are not susceptible to Lewy bodies. Neuronal cytoplasmic inclusions can also be observed in the substantia nigra, cingulate cortex, amygdala, hippocampus, entorhinal cortex, hypothalamus and neocortex [159, 160]. Purkinje cells in the cerebellum do not have neuronal cytoplasmic inclusions, but abundant α-synuclein oligomers can be detected by proximity ligation assay [161]. Some MSA cases may have concurrent Lewy body disease [148]. In such cases, Gallyas silver staining is useful in distinguishing neuronal cytoplasmic inclusions from Lewy bodies; the former is positive, but the latter is not [162]. The clinicopathologic significance of neuronal cytoplasmic inclusions has been reviewed by Cykowski and colleagues in a large cohort of MSA cases. They found that neuronal cytoplasmic inclusions in neocortex were associated with cognitive impairment [160]. Other studies have reported that the burden of neuronal cytoplasmic inclusions is associated with cognitive impairment or memory loss in MSA [43, 44, 163].

### 4.5 Atypical subtypes of MSA

Although the diagnostic criteria for MSA require neurodegeneration in striatonigral or olivopontocerebellar systems, or both, some MSA cases do not have significant neurodegeneration even though they have widespread GCI. This rare subtype is referred to as “minimal change” MSA [164–168]. Cases of minimal change MSA suggest that GCI formation precedes neuronal loss. Clinical presentations of minimal change MSA vary. Some patients are asymptomatic (“preclinical MSA”) [165, 168], but in a case series from the UK, all minimal change MSA patients had respiratory dysfunction and early orthostatic hypotension [166]. Rarely, minimal change MSA has been reported with a limbic-predominant distribution of α-synuclein pathology [167].

MSA was previously considered three distinct disorders. Clinical presentations of each disorder have been considered typical features of MSA (i.e., autonomic dysfunction [Shy-Drager syndrome], parkinsonism [MSA-P], and cerebellar ataxia [MSA-C]). Several case series, however, have reported atypical clinical presentations of MSA [169–172]. Aoki and colleagues coined the term “frontotemporal lobar degeneration (FTLD)-synuclein” for a rare subtype of MSA, based on a series of four patients with atypical MSA [173]. These patients had clinical features consistent with frontotemporal dementia, including corticobasal syndrome, progressive nonfluent aphasia, or behavioral variant frontotemporal dementia. FTLD-synuclein cases had widespread GCI as well as striatonigral degeneration of MSA. Furthermore, abundant neuronal cytoplasmic inclusions, including Pick body-like inclusions, and severe neuronal loss were observed in limbic structures and the neocortex (Fig. 6I) [173].

More recently, Ando and colleagues reported that 12 of 146 (8%) of MSA had abundant neuronal cytoplasmic inclusions in the hippocampus (Fig. 6 J) and parahippocampal gyrus (“hippocampal MSA”) [174]. Severe neuronal loss with gliosis in the hippocampus and the medial temporal atrophy were also observed. Patients with hippocampal MSA had a longer disease duration and higher prevalence of cognitive impairment compared to typical MSA, but they lacked clinical features of FTLD-synuclein [174]. Despite differences in clinical presentations, hippocampal MSA and FTLD-synuclein share pathologic features; therefore, a subset of MSA may have a vulnerability to limbic structures, and these subtypes can be considered to belong to the same subtype of MSA.

### 4.6 α-Synuclein pathology in the peripheral nervous system

Lewy bodies are widely distributed not only in the central nervous system, but also in the peripheral autonomic nervous system, including peripheral nerves and neurons in the autonomic ganglia of the heart, submandibular glands, enteric nervous system, adrenal glands, skin and skin adnexa [175–183]. Therefore, detection of Lewy bodies in the peripheral tissues has been investigated as a promising diagnostic tool for Lewy body disease. Immunohistochemistry for phosphorylated-α-synuclein has detected Lewy bodies in biopsy samples of skin, enteric nervous system and other organs, although the sensitivity and specificity vary among studies probably due to different protocols (e.g., biopsy sites, antibodies, thickness of the sections, etc.) [184, 185]. One of the earliest studies by Ikemura and colleagues performed immunohistochemistry using skin samples from the arm and abdomen, showing 70% sensitivity in PD and PDD, and 40% in DLB with 100% specificity [177]. In contrast, Beach and colleagues did not detect phosphorylated-α-synuclein histopathology of the abdominal skin and scalp in patients with PD, DLB, incidental Lewy body disease, or Alzheimer’s disease with Lewy bodies, as well as healthy individuals (0% sensitivity and 100% specificity) [180]. Doppler and colleagues reported phosphorylated-α-synuclein-positive fibers of the subepidermal plexus and dermal nerve bundles not only in PD (73%), but also in MSA (75%) with 100% specificity [186]. Notably, the distribution of α-synuclein deposits was different between PD and MSA [186–188]; phosphorylated-α-synuclein was detected in autonomic fibers in PD; whereas, it was mainly detected in unmyelinated somatosensory fibers in MSA. Therefore, skin biopsy with phosphorylated-α-synuclein immunohistochemistry can be used for distinguishing two diseases. Interestingly, phosphorylated-α-synuclein deposits in cutaneous autonomic nerves can be detected by immunohistochemistry in 56–82% of patients with isolated RBD, a prodromal phase of synucleinopathies [189–193]. The presence of α-synuclein was associated with greater autonomic dysfunction in isolated RBD [192]. The results suggest that evaluation of peripheral nervous system involvement might be a feasible biomarker even in the prodromal phase of synucleinopathies. Increasingly sensitive and specific methods to detect abnormal α-synuclein in peripheral tissues (e.g., proximity ligation assay, real-time quaking-induced conversion [RT-QuIC], or protein misfolding cyclic amplification [PMCA]; see Sect. 5.4) offer hope for peripheral biomarkers for the disease [194–196].

Lewy-related pathology in the peripheral nervous system is not only useful as a promising diagnostic marker, but also important in considering the pathogenesis of Lewy body disease. Braak and colleagues hypothesized that Lewy-related pathology begins in the enteric nervous system and retrogradely propagates to the dorsal motor nucleus of the vagus through the vagal nerve [176]. Several studies using rodent models support this hypothesis [197–199]. Uemura and colleagues inoculated α-synuclein preformed fibrils into the stomach wall of wild-type mice and were able to detect Lewy body-like α-synuclein-positive aggregates in the dorsal motor nucleus of the vagus after variable time intervals [199]. This vagal nucleus α-synuclein pathology was abolished by vagotomy performed before inoculation of α-synuclein fibrils. This provides strong support for retrograde transport of inoculated α-synuclein via the vagus nerve. On the other hand, a study of 466 whole-body autopsies did not find any cases with Lewy-related pathology in the peripheral nervous system without concomitant involvement of the central nervous system, arguing against Braak’s hypothesis [200]. The relation of Lewy-related pathology in the peripheral and central nervous systems remains elucidated [28].

## 5. Distinct α-synuclein strains in Lewy body disease and MSA

### 5.1 Distinct seeding activity

As noted above, synucleinopathies are clinically and pathologically heterogeneous, but it remains unknown how the same protein can be associated with distinct pathologies (e.g., Lewy bodies and GCI) and what factors determine neuroanatomical and cell type vulnerability. Accumulating evidence suggests that distinct α-synuclein strains may be associated with different disorders. Several studies using α-synuclein extracted from MSA and Lewy body disease showed distinct seeding activities *in vitro* and *in vivo* [201–204]. Prusiner and colleagues reported that extracts from MSA brains, but not from PD brains, induced aggregation of α-synuclein in cultured cells expressing YFP-tagged A53T-mutated human α-synuclein [201]. Similarly, Woerman and colleagues demonstrated that α-synuclein from MSA postmortem brains induced protein aggregations in cultured cells, whereas α-synuclein isolated from Lewy body disease had no effect [202]. Yamasaki and colleagues also demonstrated distinct biochemical properties of α-synuclein from MSA and PD using a FRET biosensor assay based on expression of A53T α-synuclein-CFP/YFP [203]. Both soluble and insoluble fractions of MSA had robust seeding activity, while only insoluble fractions of PD had seeding activity. Moreover, the morphology of induced cellular inclusions was different in MSA and PD. Peng and colleagues demonstrated distinct seeding activity of brain-derived α-synuclein fibrils from GCI and Lewy bodies [204]. They treated primary oligodendrocytes that expressed α-synuclein with GCI-derived α-synuclein and Lewy body-derived α-synuclein and found that GCI-derived α-synuclein was 1,000 times more potent at seeding compared with Lewy body-derived α-synuclein. Injection of GCI-derived α-synuclein induced abundant neuronal inclusions in wild-type mice, but Lewy body-derived α-synuclein did not induce neuronal inclusions at 3 months after the injection. These experiments indicated that α-synuclein derived from GCI had more potent seeding activity *in vitro* and *in vivo* than that derived from Lewy bodies, which may correlate with a more aggressive disease course in MSA compared to Lewy body disease [204]. Disease-specific seeding activity may reflect conformational differences of α-synuclein fibrils [204].

### 5.2 Distinct conformation of α-synuclein with cryo-EM

**Fig. 7:**
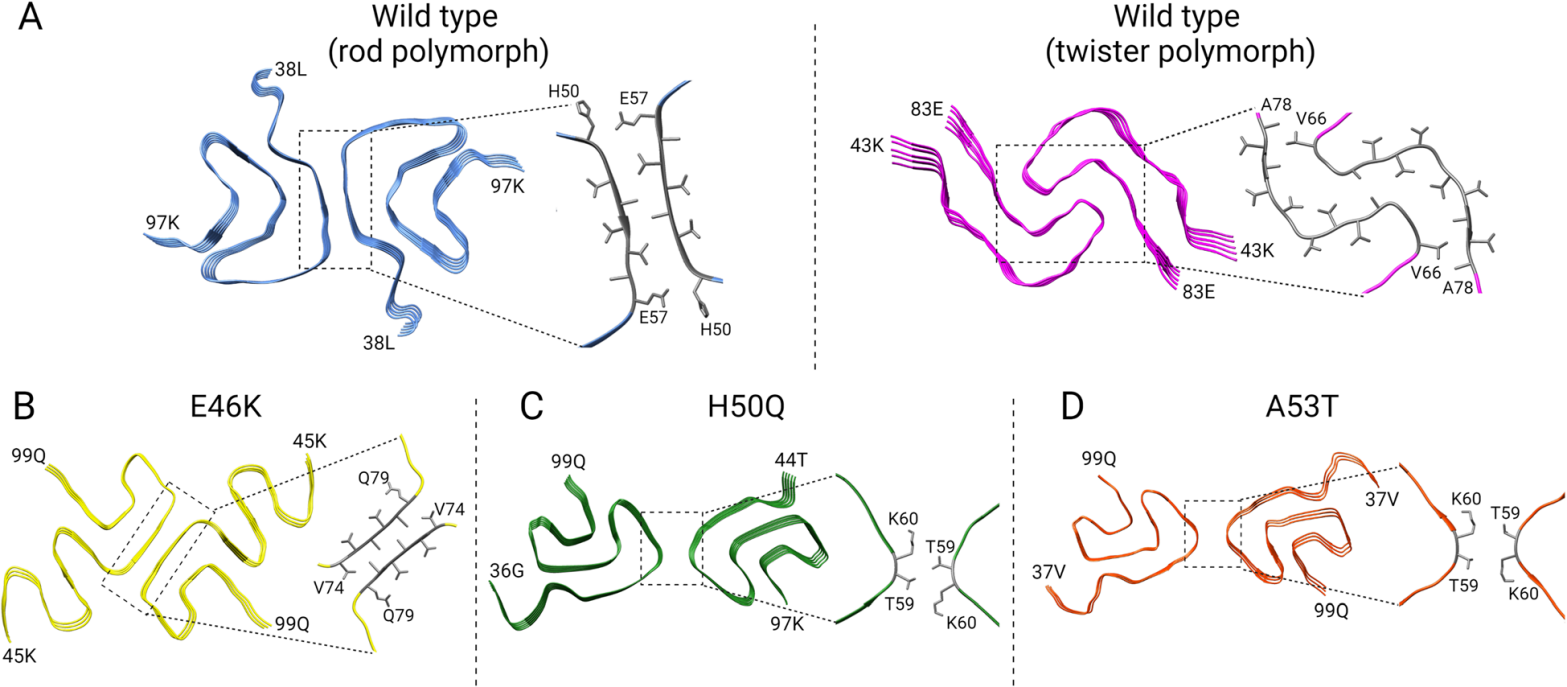
The cryo-EM structures of recombinant α-synuclein fibrils. **A** Two polymorphs of filaments from wild type fibrils: rod (Protein Data Bank [PDB] ID: 6CU7) and twister (PDB ID: 6CU8). **B** E46K mutant fibrils (PDB ID: 6L43). **C** H50Q mutant fibrils (PDB ID: 6PES). **D** A53T mutant fibrils (PDB ID: 6LRQ). Dotted boxes indicate the inter-protofilament interfaces of each α-synuclein fibril

Cryo-EM has emerged in the last decade as an effective tool for structure determination [205]. The advancement of transmission electron microscope optics and software for data analysis enables three-dimensional reconstruction of macromolecular assemblies at near-atomic resolution. Several studies using cryo-EM have revealed multiple polymorphs from recombinant α-synuclein [206–210]. Li and colleagues investigated full-length recombinant human α-synuclein and determined two predominant polymorphs, which they termed “rod” and “twister.” Both polymorphs were composed of two protofilaments with highly conserved kernel structures, but they differed in inter-protofilament interfaces [207]. The interface between the two protofilaments in the rod polymorph contained residues H50-E57 from the preNAC region (Fig. 7 A), while the interface in the twister polymorph contained residues V66-A78 from the NACore. Notably, six missense mutations (p.E46K, p.H50Q, p.G51D, p.A53E, p.A53T and p.A53V) that cause familial PD are located in the preNAC steric zipper (i.e., residues 46-56) in the rod polymorph. This suggests that PD-associated mutations may disrupt the preNAC zipper of fibril cores in the rod polymorph, while having little impact on the stability of the twister polymorph.

Recent cryo-EM studies using recombinant full-length α-synuclein fibrils with missense mutations have provided more direct evidence on how these mutations affect the conformation of α-synuclein fibrils. N-terminally acetylated p.E46K mutant α-synuclein fibrils had conformational changes in the N-terminal region of the fibril core [211]. The protofilament interface of p.E46K mutant fibril covered V74-Q79, which differed from the interface covering H50-E57 in type 1 polymorph (Fig. 7B). The p.H50Q mutation was associated with two new polymorphs: narrow fibrils and wide fibrils [209]. The narrow fibrils were formed from a single protofilament (protofilament A), whereas the wide fibrils were composed of two protofilaments (protofilaments A and B). The inter-protofilament interface of the wide fibrils had only T59 and K60 (Fig. 7 C). N-terminally acetylated p.A53T mutant α-synuclein fibrils also had a small protofilament interface, consisting of T59 and K60 with no obvious interaction (Fig. 7D) [212]. The protofilament interface of the mutant fibril was less stable than the wild type. These results indicate that missense mutations associated with familial PD can alter the conformation of protofilaments and inter-protofilament interfaces, resulting in variable α-synuclein fibrils with distinct aggregation kinetics, seeding activity, and cytotoxicity.

**Fig. 8:**
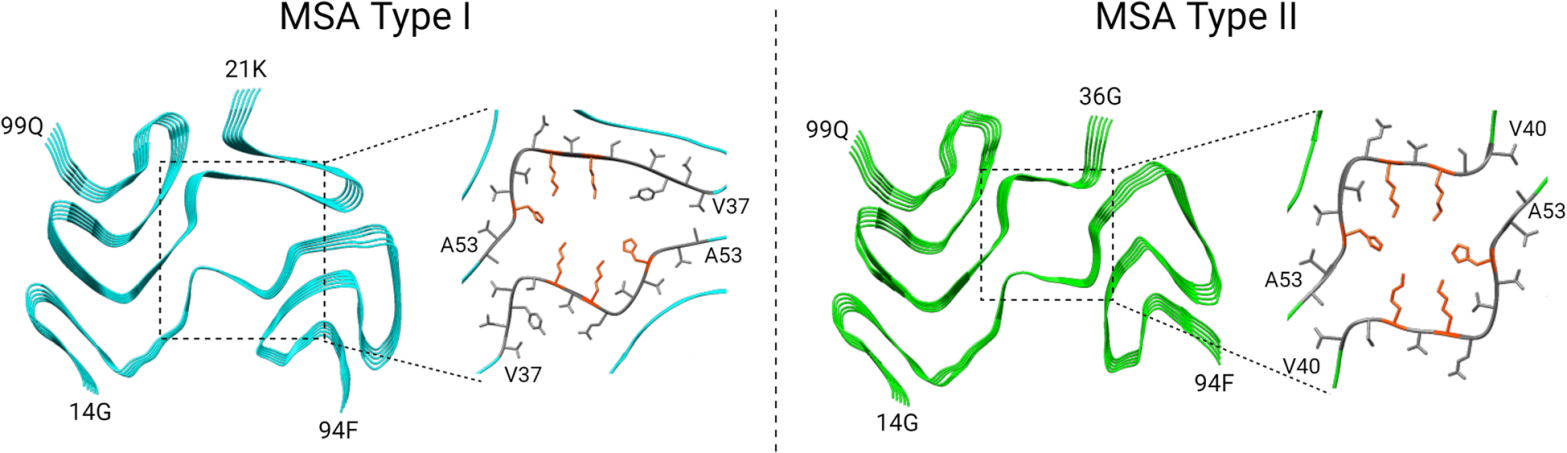
The cryo-EM structures of α-synuclein fibrils from MSA brains. Two distinct filaments: type I (PDB ID: 6XYO) and type II (PDB ID: 6XYP). Dotted boxes indicate the inter-protofilament interfaces. 43 K, 45 K, and 50 H are shown in red

Although many studies have used recombinant α-synuclein fibrils, evidence suggests that structures of recombinant filaments assembled *in vitro* may be different from those derived from human brains, as has been observed with tau protein [213–215]. Only a few studies have been reported on α-synuclein derived from human brains. One such study determined the atomic structures of α-synuclein fibrils isolated from MSA brains [216], in which two different types of filaments (type I and type II filaments) were observed (Fig. 8). The ratio of type I to type II differed among MSA patients. Both filaments had two different protofilaments, which consisted of an extended N-terminal arm and a compact C-terminal body. Unlike recombinant α-synuclein fibrils, both filaments were asymmetric. The inter-protofilament interface of type I fibrils contained V37-A53, while that of type II contained V40-A53. Mutations in p.G51D and p.A53E, which cause atypical synucleinopathies with features of PD and MSA [77, 78, 217], are located in this interface. These mutations increase the negative charge around the central cavity, which may lead to their different molecular composition [216]. The interface between the two different protofilaments forms a large cavity surrounded by side chains of K43, K45 and H50 from each protofilament (Fig. 8). This cavity encloses non-proteinaceous molecules. Studies by Puentes and colleagues using computational chemistry hypothesized that non-proteinaceous density in α-synuclein fibrils may be poly(ADP-ribose) (PAR), a negatively charged polymer generated by PAR polymerase-1 [218]. Previous studies have shown that PAR binds to α-synuclein and accelerates α-synuclein fibrillization, which results in cell death via parthanatos [219]. Using proximity ligation assay, they demonstrated PAR-α-synuclein interactions in postmortem brain tissue from PD, PDD, and MSA [218]. Furthermore, they confirmed that PAR and α-synuclein interact via electrostatic forces involving positively charged lysine residues in α-synuclein [218].

Interestingly, the same group who reported the cryo-EM structures of α-synuclein filaments from MSA brains investigated whether seeded assemblies of α-synuclein had the same structures as brain-derived seeds [220]. They seeded the *in vitro* assembly of recombinant wild-type human α-synuclein with α-synuclein derived from MSA brains using PMCA. The resultant filaments showed distinct conformations from the original MSA brain-derived seed, indicating that the products from *in vitro* seeding do not necessarily replicate the atomic structure of the seed.

### 5.3 Machine learning-based protein structure prediction

Although advances in cryo-EM enable structural determination at near-atomic resolution, this technique is labor-intensive and not high throughput. As a potential alternative approach, machine learning algorithms can contribute to protein structure predictions [221, 222]. One such algorithm, AlphaFold2, has been used to accurately predict the structures of proteins. These state-of-the-art technologies hold great promise to provide predictions of the atomic-level structure of proteins; however, limitations must be addressed in predicting the structures of amyloid proteins, such as α-synuclein. As noted above, α-synuclein has multiple polymorphs and various intermediates and aggregates; therefore, the same amino acid sequence can produce different structures, which hinder *in silico* sequence-based predictions [223]. Nevertheless, further understanding of the atomic structure of α-synuclein may elucidate mechanisms of seeding activity and contribute to designing drugs targeting specific structural features of α-synuclein.

### 5.4 α-Synuclein seeding assays as potential biomarkers

A number of studies have sought fluid biomarkers for synucleinopathies [224–230], but reliable biomarkers are not yet widely available. Recently, biomarkers directly assessing α-synuclein have been developed using RT-QuIC or PMCA. RT-QuIC is an ultrasensitive biochemical assay used to detect self-templating amyloidogenic proteins in brain tissue and cerebrospinal fluid (CSF). It has also been explored to various peripheral tissue types. It was originally developed to detect pathogenic seeding of prions [231, 232], and is currently used in diagnosis of Creutzfeldt-Jakob disease [233]. PMCA is a technology that was originally established to detect misfolded prion aggregates through sonication-based amplification of misfolding and aggregation [234, 235]. In contrast to RT-QuIC, PMCA relies on long-duration shaking instead of more rapid sonication. Both techniques have been successful as biomarkers for aggregation-prone proteins, including α-synuclein [236].

Studies using RT-QuIC or PMCA to diagnose synucleinopathies with CSF samples are summarized in Table 2 [237–249]. Early studies used brain and CSF to develop and validate the RT-QuIC assays. Fairfoul and colleagues demonstrated α-synuclein seeding activity using a small number of CSF samples, and positive reactions were observed in 11/12 autopsy-confirmed cases of pure DLB (92% sensitivity), 11/17 cases of DLB with Alzheimer’s disease (65% sensitivity), 2/13 cases of Alzheimer’s disease with incidental Lewy bodies, and 2/2 cases of PD (100% sensitivity) [237]. None of the healthy controls, 5 cases of tauopathies, and 30 cases of pure Alzheimer’s disease showed positive RT-QuIC. Thus, the overall specificity was 100%. They also investigated CSF from 20 patients with clinically diagnosed PD (95% sensitivity) and 3 patients with isolated RBD (100% sensitivity). Interestingly, α-synuclein positivity with seeding assays was detected in individuals at risk of subsequent diagnosis of PD or DLB, indicating that seeding assays may be useful in detecting prodromal α-synucleinopathies [245].

**Table 2:** Studies on α-synuclein RT-QuIC using CSF

Author	Autopsy	Disease	N	N Control	Sensitivity	Specificity
Fairfoul [237]	Yes	Pure DLB	12	20	92%	100%
		DLB with AD	17	20	65%	100%
		AD with ILBD	13	20	15%	100%
		PD	2	20	100%	100%
	No	PD	20	15	95%	100%
		IRBD	3	15	100%	100%
Manne [248]	No	PD	15	11	94%	100%
Shahnawaz [238]	No	PD	76	65	88%	94%
		DLB	10	65	100%	94%
		MSA	10	65	80%	94%
Groveman [239]	Yes	PD	12	31	92%	100%
		DLB	17	32	94%	100%
Bongianni [240]	Yes	DLB	7	49	100%	96%
		MSA	1	50	100%	96%
		Mixed LBD *^2^	20	51	90%	96%
	No	DLB	26	10	65%	100%
Kang [241]	No	PD	105	79	96%	82%
Van Rumund [242]	Yes *^1^	PD	53	52	84%	98%
		MSA	17	52	35%	98%
		DLB	1	52	100%	98%
Garrido [243]	No	PD	10	10	90%	80%
		LRRK2-PD	15	10	40%	80%
		LRRK2-NMC	16	10	19%	80%
Rossi [244]	Yes	DLB	14	101	100%	98%
		Mixed LBD *^3^	7	102	86%	98%
	No	DLB	34	166	97%	94%
		PD	71	167	94%	94%
		IRBD	18	168	100%	94%
		PAF	28	169	93%	94%
Iranzo [245]	No	IRBD	52	40	90%	90%
Shahnawaz [246]	No	PD	94	56	94%	100%
		MSA	75	56	85%	100%
		PD vs. MSA	88	65 *^4^	97%	94%
Rossi [257]	No	MCI-LB	81	58	95%	97%
Perra [249]	No	DLB	16	32	100%	90%

*1: 98% of cases are not autopsy-confirmed. *2 This includes LBD with AD (*n* = 15), LBD with PART (*n* = 2), and CJD with LBD (*n* = 3). *3 This includes CJD with DLB (*n* = 2), CJD with brainstem LBD (*n* = 3), and other primary diagnoses with limbic LBD (*n* = 1) or brainstem LBD (*n* = 1). *4 This number indicates MSA patients. The sensitivity and specificity are for PD against MSA. *Abbreviations*: *AD *Alzheimer’s disease, *CJD *Creutzfeldt-Jakob disease, *DLB *Dementia with Lewy bodies, *ILBD *Incidental Lewy body disease, *IRBD *Isolated rapid eye movement sleep behavior disorder, *LBD* Lewy body disease, *MCI *Mild cognitive impairment, *MSA *Multiple system atrophy,  *PD *Parkinson’s disease

In addition to studies comparing synucleinopathies to non-synucleinopathies [238], Shahnawaz and colleagues used PMCA to compare CSF from patients with PD and MSA [246]. They found distinct aggregation profiles in PD and MSA. Based on different kinetics of aggregation, they correctly identified PD in 85 of 88 samples (97% sensitivity) and 61 of 65 samples from MSA patients (94% sensitivity). Combining all samples, they correctly distinguished PD from MSA in 146 of the 153 samples for an overall accuracy of 95%.

Recent studies have applied RT-QuIC to biopsies of skin, submandibular gland, and olfactory mucosa (Table 3) [241, 247–252]. Abdominal skin from autopsy-confirmed patients with PD, MSA, Lewy body dementia, and non-synucleinopathies showed positive α-synuclein seeding activity in 44 of 47 PD (94% sensitivity), 2 of 3 MSA (67% sensitivity), all 7 Lewy body dementia (100% sensitivity), and 6 of 73 control group (92% specificity) [252]. This study also evaluated skin biopsy samples from living patients with PD and non-PD subjects, which showed 95% sensitivity and 100% specificity. Manne and colleagues sampled skin tissues of the scalp from patients with PD and control individuals and showed 96% sensitivity and 96% specificity [253]. More recently, a study compared the diagnostic accuracy based on RT-QuIC with α-synuclein immunofluorescence on skin biopsies from C7 paravertebral region and thigh, demonstrating that both RT-QuIC and immunofluorescence showed high diagnostic accuracy in differentiating synucleinopathies from non-synucleinopathies [256]. The submandibular gland was also used for RT-QuIC [251]. Positive seeding activity was present in all 13 cases of PD and 3 cases of incidental Lewy body disease (100% sensitivity), but only 1/16 control cases (94% specificity). Despite the high sensitivity and specificity, the invasiveness of submandibular gland biopsy procedure may limit its clinical application.

**Table 3:** Studies on α-synuclein RT-QuIC using peripheral tissue samples

Author	Tissue	Autopsy	Disease	N	N Control	Sensitivity	Specificity
De Luca [250]	OM	No	PD	18	18	56%	83%
			MSA	11	18	82%	83%
Manne [251]	SMG	Yes	PD	13	16	100%	94%
			ILBD	3	16	100%	94%
Wang [252]	Abdominal skin	Yes	PD	47	73	94%	93%
			Lewy body dementia	7	73	100%	93%
			MSA	3	73	67%	93%
	Biopsy skin *^1^	No	PD	20	21	95%	100%
Manne [253]	Frozen skin *^2^	Yes	PD	25	25	96%	96%
	FFPE skin *^2^		PD	12	12	75%	83%
Mammana [254]	Skin *^3^	Yes	PD/DLB	2	40	100%	98%
			ILBD	7	40	86%	98%
		No	DLB	15	41	100%	95%
			PD	13	41	77%	95%
Stefani [255]	OM	No	IRBD	63	59	44%	90%
			PD	41	59	46%	90%
Perra [249]	OM	No	DLB	43	38	81%	92%

*1 Biopsy skin samples are obtained from the leg or posterior cervical region. *2 The scalp skin is collected from the posterior lower occipital region along the midline. *3 Skin punches are collected from the cervical region and/or thigh. *Abbreviations*: *FFPE *Formalin-fixed paraffin-embedded, *ILBD *Incidental Lewy body disease, *IRBD *Isolated rapid eye movement sleep behavior disorder, *MSA *Multiple system atrophy, *PD *Parkinson’s disease, *OM *Olfactory mucosa, *SMG *Submandibular glands

Olfactory mucosa has also been investigated as a potential sampling site for RT-QuIC [249, 250, 254]. De Luca and colleagues reported that 10/18 cases of PD (56% sensitivity) and 9/11 cases of MSA (82% sensitivity) showed RT-QuIC seeding activity, while 16% of primary tauopathies showed positive (84% specificity) [250]. Perra and colleagues explored the diagnostic accuracy of α-synuclein RT-QuIC using the olfactory mucosa of patients with DLB, which showed 81% sensitivity and 92% specificity [249]. CSF was also analyzed in a subset of patients, resulting in 100% sensitivity and 90% specificity. Stefani and colleagues demonstrated positive α-synuclein RT-QuIC seeding activity in the olfactory mucosa in 44% of isolated RBD patients and 46% in PD patients with an overall specificity of 90% [254]. Interestingly, isolated RBD patients with positive α-synuclein seeding activity had olfactory dysfunction more frequently than those without seeding activity (79% vs. 23%). Although the sensitivity for detecting α-synuclein seeding activity was moderate in these studies [249, 250, 254], olfactory mucosa sampling by nasal swabbing is less invasive than a lumbar puncture, skin biopsy, or submandibular gland biopsy.

Although current diagnostic criteria do not include *in vitro* seeding assays as supportive biomarkers, these new methods may be involved in future criteria [23, 27, 37, 48, 257]. These assays will not only assist detection of synucleinopathies at an early stage, which is often a clinical challenge [258, 259], but also help recruit patients for future clinical trials of disease-modifying therapies targeting α-synuclein aggregation and propagation. Nevertheless, there are still some obstacles that need to be overcome in order for RT-QuIC to be a routine screening technique. First, the methods of these seeding assays, including a defined purification protocol for the substrate and sampling sites of peripheral tissues, must be standardized [260, 261]. Second, the majority of studies lack autopsy-confirmed diagnosis. Lewy body pathology can coexist with non-synucleinopathies, particularly Alzheimer’s disease; therefore, a positive result in non-synucleinopathies might not be a false-positive, but rather detected copathology. In addition, RT-QuIC and PMCA are more expensive assays compared to phosphorylated-α-synuclein immunofluorescence, which is also a promising approach to diagnose synucleinopathies as discussed in Sect. 4.6. Further studies are needed to confirm the usefulness of seeding assays and immunohistochemistry using the peripheral tissue samples for the diagnosis of synucleinopathies.

## 6. Conclusions

Synucleinopathies are clinically and pathologically heterogeneous neurodegenerative disorders for which there is currently no cure. To develop disease-modifying therapies for synucleinopathies, it is essential to elucidate how α-synuclein converts to pathologic oligomers and fibrils, which form Lewy bodies in Lewy body disease and GCI in MSA. Interaction between α-synuclein and lipid membranes (e.g., mitochondria, lysosome, synaptic vesicles, etc.) has gained traction as a part of the pathomechanism of Lewy body formation, whereas that of GCI formation remains to be elucidated. Classification and staging schemes are important to understand the clinicopathological heterogeneity of the disease. The current staging schemes of Lewy body disease are based upon evaluation only of the central nervous system. Given that Lewy body pathology is present in the peripheral nervous system, even in prodromal stages of the disease, a more comprehensive staging approach that samples peripheral tissues may provide more comprehensive staging.

The hypothesis that clinicopathologic heterogeneity of synucleinopathies is linked to different strains of α-synuclein is supported by mounting experimental evidence from seeding assays and advanced structural biology. As distinct strains of α-synuclein are increasingly used as biomarkers, diagnostic accuracy will likely improve. Although seeding assays using peripheral tissue samples hold promise, the methodologies need to be standardized for future clinical use. Accurate and early clinical diagnosis will be increasingly crucial for intervention early in the disease for future disease-modifying clinical trials.

## Data Availability

Not applicable.

## Abbreviations

cryo-EM: Cryogenic electron microscopy
CSF: Cerebrospinal fluid
DLB: Dementia with Lewy bodies
FTLD: Frontotemporal lobar degeneration
GCI: Glial cytoplasmic inclusion
GWAS: Genome-wide association study
ICDLB: International Consortium for Lewy Body Dementia
MSA: Multiple system atrophy
MSA-C: MSA with predominant cerebellar ataxia
MSA-P: MSA with predominant parkinsonism
NAC: Non-amyloid-β component
NACP: Non-amyloid component in senile plaques
OPCA: Olivopontocerebellar atrophy
OR: Odds ratio
PAR: Poly (ADP-ribose)
PD: Parkinson’s disease
PDD: Parkinson’s disease with dementia
PMCA: Protein misfolding cyclic amplification
RBD: Rapid eye movement sleep behavior disorder
RT-QuIC: Real-time quaking-induced conversion
SND: Striatonigral degeneration
STED: Stimulated emission depletion
